# Insect Cell-Based Quadrivalent Seasonal Influenza Virus-like Particles Vaccine Elicits Potent Immune Responses in Mice

**DOI:** 10.3390/vaccines12060667

**Published:** 2024-06-17

**Authors:** A. T. M. Badruzzaman, Yu-Chieh Cheng, Wang-Chou Sung, Min-Shi Lee

**Affiliations:** 1 National Institute of Infectious Diseases and Vaccinology, National Health Research Institutes (NHRI), 35 Keyan Road, Zhunan 350, Taiwan; badruzzaman.sau@gmail.com (A.T.M.B.); jessiecheng@nhri.edu.tw (Y.-C.C.); sung23@nhri.edu.tw (W.-C.S.); 2 Department of Life Sciences, National Central University, Zhongli District, Taoyuan 320, Taiwan

**Keywords:** seasonal influenza, virus-like particle (VLP), baculovirus expression system, vaccine

## Abstract

Influenza viruses can cause highly infectious respiratory diseases, posing noteworthy epidemic and pandemic threats. Vaccination is the most cost-effective intervention to prevent influenza and its complications. However, reliance on embryonic chicken eggs for commercial influenza vaccine production presents potential risks, including reductions in efficacy due to HA gene mutations and supply delays due to scalability challenges. Thus, alternative platforms are needed urgently to replace egg-based methods and efficiently meet the increasing demand for vaccines. In this study, we employed a baculovirus expression vector system to engineer HA, NA, and M1 genes from seasonal influenza strains A/H1N1, A/H3N2, B/Yamagata, and B/Victoria, generating virus-like particle (VLP) vaccine antigens, H1N1-VLP, H3N2-VLP, Yamagata-VLP, and Victoria-VLP. We then assessed their functional and antigenic characteristics, including hemagglutination assay, protein composition, morphology, stability, and immunogenicity. We found that recombinant VLPs displayed functional activity, resembling influenza virions in morphology and size while maintaining structural integrity. Comparative immunogenicity assessments in mice showed that our quadrivalent VLPs were consistent in inducing hemagglutination inhibition and neutralizing antibody titers against homologous viruses compared to both commercial recombinant HA and egg-based vaccines (Vaxigrip). The findings highlight insect cell-based VLP vaccines as promising candidates for quadrivalent seasonal influenza vaccines. Further studies are worth conducting.

## 1. Introduction

Influenza infection, more commonly known as flu, is an acute respiratory infectious disease caused by the seasonal influenza viruses. According to the World Health Organization (WHO), influenza infects 1 billion people annually and causes 3–5 million severe illness [1]. Historically, flu viruses have been associated with many deaths and hospitalizations, especially in children and elderly populations [2,3]. In the 2021–2022 flu season, the United States alone reported 470,000 to 790,000 cases, with nearly half of them requiring medical attention; the number of deaths was significant [4]. Human epidemic events are mainly caused by two types of influenza viruses: type A and type B. Based on their surface glycoproteins, hemagglutinin (HA) and neuraminidase (NA), type A viruses are further divided into many subtypes, with H1N1 and H3N2 being involved in human seasonal outbreaks [1]. On the other hand, type B viruses are further divided into two antigenically distinct lineages, namely Yamagata and Victoria [5]. Although type B viruses are not responsible for pandemics, they are potentially associated with a significant number of severe acute respiratory diseases [6]. The most effective way to confer protection against influenza viruses is through annual vaccination. Annually, WHO recommends candidate vaccine virus (CVV) strains for the southern (September) and northern (February) hemispheres based on global influenza surveillance data. Before the 2013–2014 flu season, only trivalent vaccines containing two circulating type A viruses and one type B virus, either Yamagata or Victoria, were recommended. However, many surveys have shown that human infections have been associated with the co-infection of Yamagata and Victoria lineage viruses [6]. Since the 2014–2015 flu season, WHO has released recommendations for tetravalent CVV strains, which include both circulating B lineage viruses. Current classical tetravalent virus vaccines rely mainly on embryonic chicken eggs (ECE) to produce either inactivated or live attenuated influenza vaccines. The egg-based platform has raised several concerns, including the possibility of allergic reactions to egg albumin, the time required for vaccine production, and the continuous supply of ECE during an avian influenza outbreak. Moreover, this conventional technology is sometimes associated with unintentional mutations of the virus during propagation in ECE, which may reduce vaccine efficacy [7]. Recently, advanced egg-free technologies have become attractive for the development of influenza vaccines. Non-infectious virus-like particle (VLP) technology is one of the egg-free technologies, which has several advantages over the egg-based vaccines [8]. Another problem associated with the conventional platform is the production time. In general, the ECE-based influenza vaccines take nearly six months from preparation of the CVV strain to the delivery of the ultimate vaccine to the market [9]. To address this issue, VLPs can be a great choice since they can significantly reduce the manufacturing time to four months [10], which could have a great impact for pandemic mitigations. Additionally, the baculovirus expression vector system (BEVS) we employed offers several advantages over other platforms: It yields high levels of recombinant protein, performs complex post-translational modifications, is versatile for a wide range of proteins, and has relatively lower production costs [11]. Apart from the influenza virus, many other VLP vaccines have gained market approval, especially against human papilloma virus, hepatitis B virus, hepatitis E virus, etc. [12]. Despite the favorable advantages of BEVS for VLP production, no insect cell-based quadrivalent influenza VLP vaccine co-expressing HA, NA, and M1 proteins has been developed for human use to date.

In the current study, our objective was to optimize the parameters for insect cell-based influenza VLP production using the BEVS, ensuring the expression of functional HA, NA, and M1 proteins. Finally, we compared the antibody responses of egg-based, recombinant HA, and VLP quadrivalent seasonal influenza vaccines in mice.

## 2. Materials and Methods

### 2.1. Viruses, Cell Lines, and Culture Media

Influenza viruses were cultured in MDCK cells using serum-free OptiPRO medium (ThermoFisher, St. Bend, OR, USA), and the Reed–Muench method [13] was performed to determine the 50% tissue culture infectious dose (TCID_50_). Experiments related to the live viruses were conducted in a Biosafety Level 2 facility. For cultivation of insect Sf-21 cells and High Five cells, Sf-21 cells were grown in Grace’s medium supplemented with 10% FBS, while High Five cells were cultured in ESF-921 medium (Expression Systems, Davis, CA, USA).

### 2.2. Construction of Plasmid

**Figure 1:**
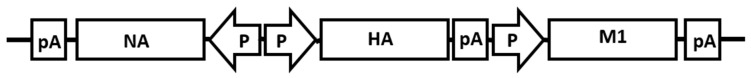
Schematic diagram of rBVs. The modified pFastBac dual vector containing three promoters was used for the expression of HA, NA, and M1 genes. NA was under the control of polyhedrin promoter and HA and M1 gene under the control of p10 promoter.

Three essential genes, HA, NA, and M1, were amplified by PCR from the cDNA of the strains listed in Table 1 and cloned into a modified pFastBac Dual plasmid (ThermoFisher, St. Bend, OR, USA) [14]. The recombinant baculoviruses (rBVs) were generated through the Bac-to-Bac baculovirus expression system according to the manufacturer’s protocol [15]. The scheme for constructing the recombinant baculoviruses is shown in Figure 1.

### 2.3. Production of Recombinant Baculovirus (rBV) and Virus-like Particles (VLPs)

Sf-21 cells (Invitrogen, Norristown, PA, USA) were cultured in Grace’s insect basal medium (Invitrogen, Norristown, PA, USA) supplemented with 10% FBS (Gibco, Norristown, PA, USA) and were transfected with bacmid DNA to generate the rBV. The virus titer of the rBV was determined by plaque assay in Sf21 cells. To produce the VLPs, High Five cells were suspension-cultured at 27 °C in serum-free ESF-921 medium (Expression System, Pittsburgh, PA, USA) at a density of 2 × 10^6^ cells/mL, followed by rBV infection with a multiplicity of infection ranging from 1 to 5. VLPs were harvested at different time points to monitor the cell viability and HA protein production by measuring HA activity in the supernatant and pellet. After harvesting, the cell debris was removed by low-speed centrifugation, and the supernatant was collected and stored at 4 °C until purification.

### 2.4. Purification of the VLPs

The influenza VLP in supernatant was pelleted by ultracentrifugation (135,000× *g* for 4 h at 4 °C) using the sucrose cushion method. The sedimented VLPs were re-suspended in the phosphate-buffered saline (PBS) with or without trehalose (final concentration is 20%).

### 2.5. Hemagglutination Assay

The hemagglutination (HA) assay was carried out by following the WHO guideline [16]. In brief, VLPs were subjected to a 2-fold serial dilution in PBS within a 96-well plate. These diluted VLPs were mixed with equal volume of 0.5% suspension of turkey red blood cell (TRBC) then incubated to room temperature (RT) for 40 min. The HA titer was determined as the maximum dilution factor of the VLPs that entirely agglutinated the TRBC.

### 2.6. Western Blot and SDS-PAGE Analysis

The presence of the desired three proteins (HA, NA, and M1) in the purified H1N1, H3N2, Yamagata, and Victoria VLPs was confirmed through Western blot analysis. To detect proteins in type A VLPs, primary antibodies including anti-HA antibodies against A/Guangdong-Maonan/SWL1536/2019 (NIBSC no. 19/314, National Institute for Biological Standards and Control, Potters Bar, UK) for H1N1, A/Hong Kong/2671/2019 (NIBSC no. 19/316, National Institute for Biological Standards and Control, Potters Bar, UK) for H3N2 HA protein, anti-NA antibodies for A/WSN/1933 (GTX125974, GeneTex, Hsinchu City, Taiwan) for H1N1, and anti-NA2 antibody (SinoBiological, 40017-T60, Taipei City, Taiwan) for H3N2 NA protein were used. For M1 protein detection in both H1N1 and H3N2 subtypes, anti-M1 antibodies specific to type A (GTX127356, GeneTex, Hsinchu City, Taiwan) were utilized. In the case of type B VLPs, anti-HA antibodies for B/Phuket/3073/2013 (NIBSC no. 14/248, National Institute for Biological Standards and Control, Potters Bar, UK) and B/Washington/02/2019 (NIBSC no. 19/218, National Institute for Biological Standards and Control, Potters Bar, UK) were used to detect the HA proteins of Yamagata and Victoria VLPs, respectively. Anti-NA antibodies (GTX128540, GeneTex, Hsinchu City, Taiwan) and anti-M1 antibodies (GTX128537, GeneTex, Hsinchu City, Taiwan) for influenza B were used to detect the NA and M1 protein in both lineages, respectively. All primary antibodies were diluted (1:4000, *v*/*v*) and incubated at RT for 1 h. Subsequently, the membrane was washed with phosphate-buffered saline with Tween 20 (PBST) and incubated at RT for 1 h with HRP conjugated secondary antibodies (1:10,000, *v*/*v*). Anti-sheep/goat IgG-HRP (Merck, Rahway, NJ, USA) was used for HA proteins, while anti-rabbit IgG-HRP (GTX213110-01, GeneTex, Hsinchu City, Taiwan) was employed for NA and M1 proteins. The HA, NA, and M1 proteins on the membrane were visualized using an imaging system (Amersham Imager 600, Amersham, Buckinghamshire, UK). To account for glycosylation, SDS-PAGE was performed for the VLP samples under both glycosylated and deglycosylated conditions by running the samples on a NuPAGE gel (ThermoFisher, St. Bend, OR, USA) followed by staining with Colloidal Blue stain (Invitrogen, Norristown, PA, USA). The relative HA, NA, and M1 protein compositions were quantified by gel analyzer software (TotalLab, Gosforth, UK).

### 2.7. Single Radial Immunodiffusion Assay (SRID)

The HA protein content of the VLPs samples and comparator vaccine antigens was determined by the standard single radial immunodiffusion (SRID) assay protocol described previously [17]. In brief, the procedure involved the initial incubation of VLP samples, commercial vaccines, and HA standards with a 1% Zwitterionic solution for 30 min. Subsequently, the diffusion took place in a 1% agarose gel that contained the appropriate standard anti-HA serum. It is crucial to note that the specific HA standard antigens and serum for A/H1N1, A/H3N2, B/Yamagata, and B/Victoria strains were provided by the National Institute for Biological Standards and Control (NIBSC).

### 2.8. Total Protein and HA Protein Quantification

The total protein concentration in the purified VLPs and commercial vaccine group used in this study was quantified by modified Lowry assay (ThermoFisher, St. Bend, OR, USA) according to the manufacturer’s protocols.

### 2.9. Transmission Electron Microscopy (TEM)

Purified VLPs samples were absorbed by floatation onto freshly discharged carbon coated grid for 2–3 min. Grids were washed with dH_2_O, then subsequently negatively stained with 1% phosphotungstic acid for 1–2 min, and the excess stain was removed. Finally, grids were air-dried and visualized using a Hitachi H-7650 transmission electron microscope (Hitachi Ltd., Tokyo, Japan) at magnifications of 15,000× and 50,000×.

### 2.10. Mice Immunization

**Figure 2:**
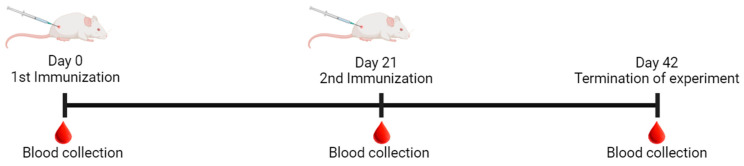
Timeline for immunization and blood collection. Female BALB/c mice (6–8 weeks of age) were vaccinated at day 0 and day 21 with either a quadrivalent insect cell-derived VLP vaccine, a recombinant HA protein vaccine (Flublok), an inactivated split virus vaccine (Vaxigrip), or PBS.

Six- to eight-week-old female SPF BALB/c mice (BALB/cAnNCrl) were obtained from BioLASCO Taiwan (Charles River’s technology licensee) and housed in the animal facility of the National Health Research Institutes (NHRI). Mice (n = 5) were immunized intramuscularly (i.m.) with ~0.5 µg HA/strain of quadrivalent VLP or commercial vaccine (either Flublok or Vaxigrip) on day 0 and day 21 (Figure 2). Commercial vaccine strain-specific HA concentrations were determined using SRID assay, and the mean HA concentration values were used for calculating the targeted immunization dose (Table 2). The mock group was immunized with PBS. Serum samples were collected on days 0, 21, and 42, as illustrated in Figure 2. All animal experimental procedures were conducted according to the IACUC-approved protocol (NHRI-IACUC-110043-A).

### 2.11. Hemagglutination Inhibition (HAI) Titers

At different time points, blood samples were collected from the mice using facial vein puncture and placed into serum separation tube (BD Microtrainer, BD, Tukwila, WA, USA), as previously described [18]. The blood samples underwent centrifugation at 3500 rpm for 10 min, and the serum was collected from supernatant. Following heat inactivation at 56 °C for 30 min, the serum samples were stored at −20 °C for subsequent analysis. To determine hemagglutination inhibition (HAI) titers, serum samples underwent processing in accordance with the guidelines set forth by WHO, as outlined in a previous study [19]. In brief, the serum samples were treated with Receptor Destroying Enzyme from Sigma-Aldrich at a 1:4 ratio, allowing for an overnight incubation at 37 °C. The serum dilution process commenced with an initial 1:10 dilution. After that, the diluted serum was mixed with eight hemagglutination units (HAU) of the corresponding inactivated viruses and incubated at RT for 30 min. Following this, the mixtures were incubated with a 0.5% suspension of TRBC at RT for 40 min. The HAI titer was determined as the equal of the maximum dilution that exhibited inhibition of HA activity. If no inhibition was detected at the starting serum dilution (1:10), the HAI was recorded as <10, and an HAI titer of 5 was employed for GMT calculation.

### 2.12. Virus Neutralization (NT) Assay

Virus neutralization (NT) assay was carried out using a modified protocol based on the manual on Animal Influenza Diagnosis and Surveillance published by WHO [16], as previously described by Chia et al. [19]. Briefly, serum samples were subjected to serial 2-fold dilution, starting from an initial dilution of 1:10. These diluted sera were then incubated with 2000 TCID_50_/_mL_ live virus at 35 °C for 2 h. The serum–virus mixture was subsequently added to a 96-well plate with monolayer of MDCK cells. Following 4 to 5 days of incubation, cytopathic effects were examined to determine serum neutralizing antibody titers. Titers were stated as equal of the maximum serum dilution that showed less than 50% of virus induced cytopathic effects.

### 2.13. Statistical Analysis

Prism 8.4.3 software (GraphPad Co., San Diego, CA, USA) was used for statistical analysis. To assess the statistical differences in the GMT among different vaccine groups, we employed an analysis of variance (ANOVA). Statistical significance was expressed as *p*-values less than 0.05 (*p* < 0.05).

## 3. Results

### 3.1. Expression and Characterization of Type A and Type B VLPs

**Figure 3:**
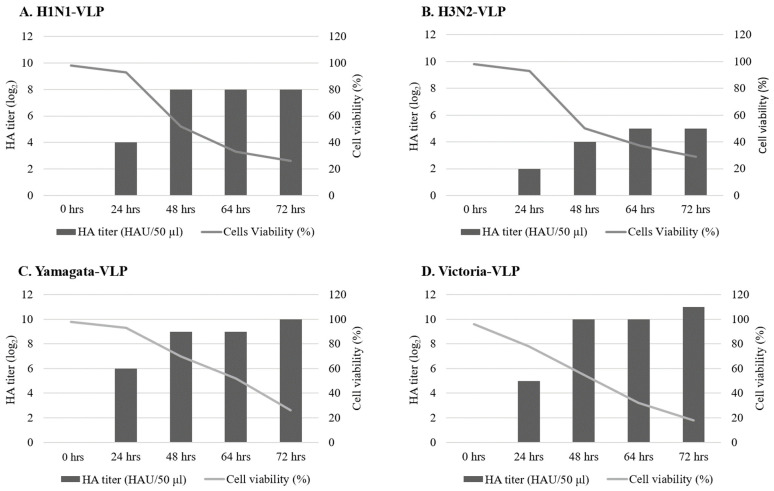
Production of different influenza VLPs in High Five cells. VLPs were harvested at different time points, and HA protein function was assessed by HA assay.

In this study, we successfully utilized an insect cell-based baculovirus expression system to generate rBVs bearing influenza HA, NA, and M1 genes from four seasonal influenza vaccine strains. Then, we produced influenza VLPs by infecting High Five cells with relevant rBVs. Viability of infected cells and HA activities were determined at different time points (Figure 3). During the production of H1N1 and H3N2 VLPs, HA activity peaked at 256 HAU/50 µL and 32 HAU/50 µL at 48 h and 64 h post infection, respectively. At this time, cell viability decreased from the initial 95% to approximately 50% and 40%, respectively. Both Yamagata and Victoria VLPs exhibited the highest HA activity at 72 h post infection, reaching 1024 HAU/50 µL and 2048 HAU/50 µL, respectively. Concurrently, cell viability declined to approximately 25% and 18% from the initial level (Figure 3). No HA activity was detected in uninfected control cells.

**Figure 4:**
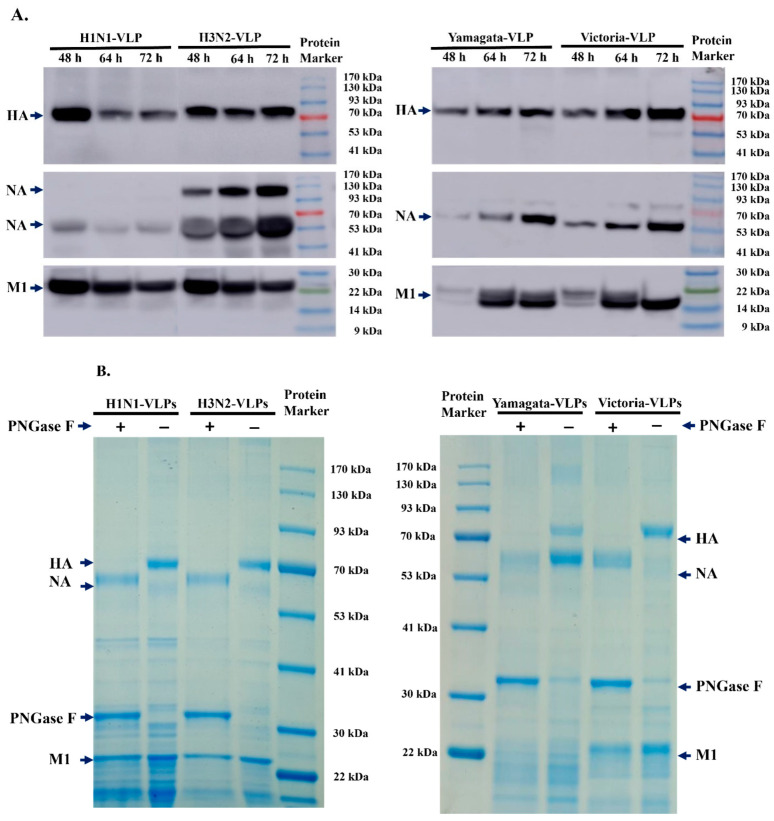
HA, NA, and M1 protein expressions of H1N1-VLP, H3N2-VLP, Yamagata-VLP, and Victoria-VLP at different time points in insect cells were detected using Western blot (**A**). Purified VLPs protein were separated by SDS-PAGE and visualized by Colloidal Blue staining. Arrows indicate HA, NA, PNGase F, and M1 proteins (**B**).

After harvesting the VLPs, we clarified the supernatant and subsequently purified them using ultra-centrifugation. The presence of the desired proteins was confirmed by Western blot. As expected, HA, NA, and M1 proteins were observed at around 70 kDa, 53 kDa, and 22 kDa, respectively (Figure 4A). The desired protein composition was also confirmed using SDS-PAGE with Colloidal Blue-stained gel analysis (Figure 4B).

**Figure 5:**
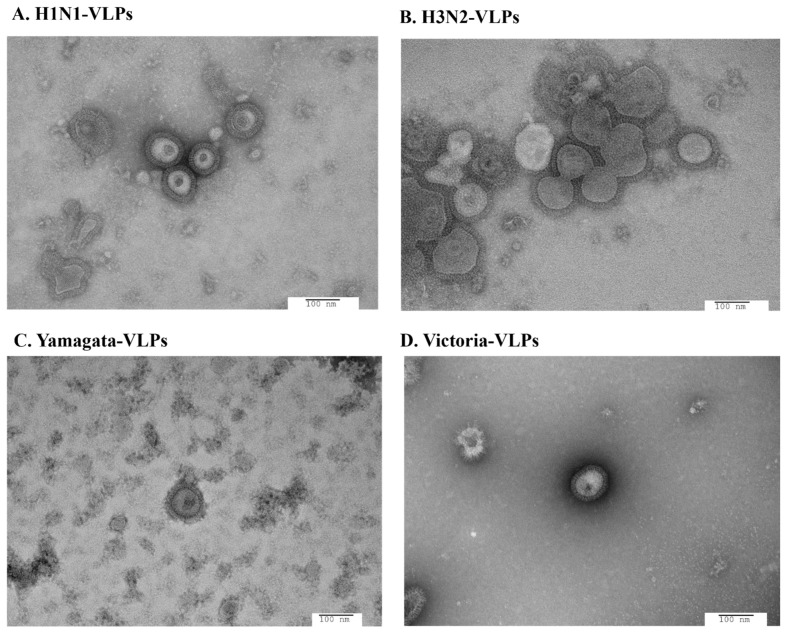
Transmission electron microscopy images of influenza VLPs prepared in High Five cells using a baculovirus expression system. Recombinant H1N1-VLPs (**A**), H3N2-VLPs (**B**), Yamagata-VLPs (**C**), and Victoria-VLPs (**D**) were morphologically like the wild-type influenza viruses and can be found as individual particles (magnification: 50,000×; scale bar: 100 nm).

Morphological characteristics of the purified VLPs were observed using TEM (Figure 5). In TEM observation, VLPs were found to be spherical particles with spikes, ranging from 100–180 nm, which is like authentic influenza viruses. These results suggest that A/H1N1, A/H3N2, B/Yamagata, and B/Victoria VLPs contain HA, NA, and M1 proteins, which are phenotypically and structurally similar to authentic influenza virions. Overall, HA activity is not a suitable marker for monitoring production of H3N2 VLPs.

We then determined the HA productivity of the seasonal VLPs by assessing HA titers through HA assay, HA protein content via SRID assay, and total protein concentration through modified Lowry assay (Table 3). After purification, SRID data revealed varying levels of HA productivity among four different influenza VLPs, including 6.6 mg/L for H1N1, 7.8 mg/L for H3N2, 2.4 mg/L for B/Yamagata, and 10.6 mg HA/L for B/Victoria. HA protein compositions of the four VLP antigens were all over 18% (Table 3); thus, all passed the requirement (≥16.6%) for influenza virus vaccines [20].

### 3.2. Pilot Evaluation of VLPs Stability

The stability of influenza VLP antigens was evaluated by HA and SRID assays, which showed that VLPs were gradually degraded in PBS within three months (Table 4). Therefore, the effect of commonly used stabilizers (trehalose compounds, 20% *v/v* in PBS) was evaluated. VLPs were mixed with trehalose and stored for up to 12 months at 4 °C and −20 °C. HA activity and HA protein concentration were measured as shown in Table 5. All the VLPs maintained most of the HA protein concentration over the study period, showing HA concentration decreased less than 20% after 12 months of storage (Table 5). The best results were obtained for the formulations that were stored at 4 °C; over 80% of the VLPs were still intact after 12 months.

### 3.3. Immunogenicity of Quadrivalent VLPs in Mice

**Figure 6:**
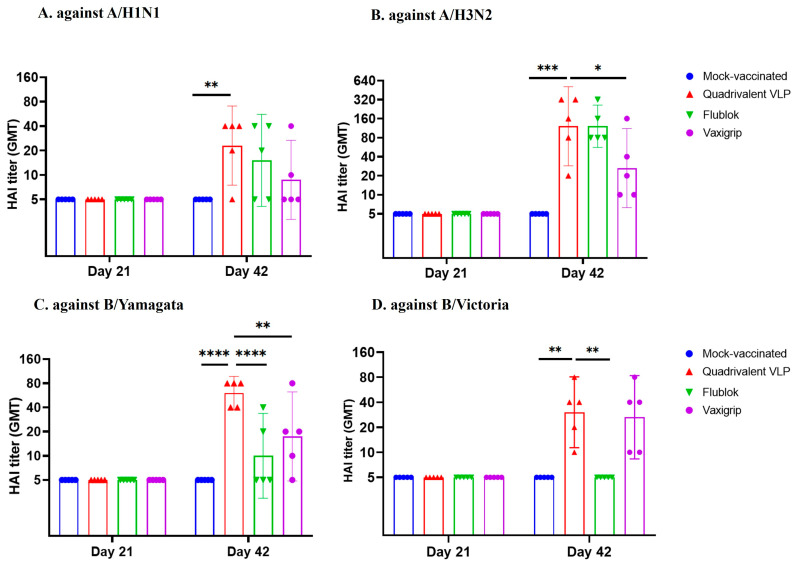
Serum HAI antibody titers (GMT with 95% CI) against homologous A/H1N1 (A/Hawaii/70/2019), A/H3N2 (A/Minnesota/41/2019), B/Yamagata (B/Brisbane/09/2014), and B/Victoria (B/Darwin/7/2019) strains in mice immunized with quadrivalent VLPs, Flublok, and Vaxigrip vaccine antigens. Statistical analysis (two-way ANOVA) was conducted with multiple comparisons using the Tukey test. *, *p* < 0.05; **, *p* < 0.01; ***, *p* < 0.001; ****, *p* < 0.0001; no asterisk/ns, *p* > 0.05.

In the mouse study, we utilized a quadrivalent formulation of VLPs, encompassing similar dosages (~0.5 µg) of A/H1N1, A/H3N2, B/Yamagata, and B/Victoria VLPs. Our objective was to assess the potential of quadrivalent VLPs as a promising candidate for a future influenza vaccine. We included two commercially available vaccines for comparison: the recombinant protein-based vaccine Flublok and the egg-based inactivated virus vaccine Vaxigrip. As part of our investigation, we conducted an analysis of HAI titers in serum samples collected from immunized subjects at 21 days after the first dose (day 21) and second dose (day 42). Notably, none of the vaccinated groups exhibited HAI titers after the initial immunization, but HAI antibodies were observed following the second dose (Figure 6).

**Figure 7:**
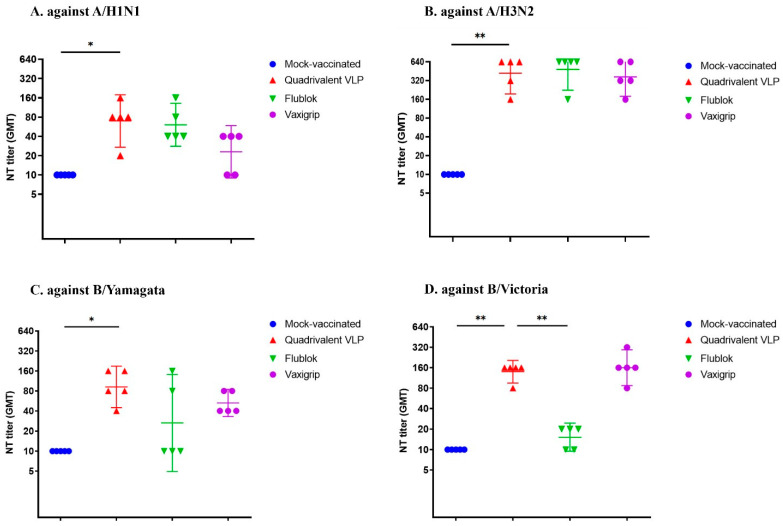
Serum NT antibody titer (GMT with 95% CI) in vaccinated mice after three weeks of booster immunization against homologous A/H1N1 (A/Hawaii/70/2019), A/H3N2 (A/Minnesota/41/2019), B/Yamagata (B/Brisbane/09/2014), and B/Victoria (B/Darwin/7/2019) viruses. Statistical analysis (one-way ANOVA) was conducted with multiple comparisons using the Tukey test. *, *p* < 0.05; **, *p* < 0.01; no asterisk or ns, *p* > 0.05.

Mice that received two doses of the quadrivalent VLP vaccine demonstrated a significant increase in serum HAI titers against the corresponding antigens A/H1N1, A/H3N2, B/Yamagata, and B/Victoria strains (geometric mean titer (GMT): 22.97, 121.26, 60.63, and 30.31, respectively). Surprisingly, two doses of the Flublok vaccine could induce significant HAI titers only for A/H1N1 and A/H3N2 (GMT: 15.16, and 121.26, respectively) but not for B/Yamagata (GMT: 10) or B/Victoria (GMT: 5). Interestingly, two doses of the egg-based Vaxigrip vaccine could generate significant HAI antibody titers only for A/H3N2, B/Yamagata, and B/Victoria (GMT: 26.39, 17.41, and 26.39, respectively) but not for A/H1N1 (GMT: 8.71) (Figure 6). Similar findings for the neutralizing (NT) antibody response were observed. We found the Flublok and Vaxigrip groups induced similar NT antibody titers against influenza H1N1, H3N2, and B/Yamagata but not B/Victoria (Figure 7).

Overall, both the VLP group and the Vaxigrip group consistently exhibited comparable antibody (HAI and NT) responses for four vaccines strains, but the Flublok group demonstrated lower antibody responses against the B strains (Figure 6 and Figure 7).

## 4. Discussion

Seasonal influenza remains a significant global health concern, necessitating the development of effective and adaptable vaccine production platforms. This study investigated the potential of utilizing BEVS to produce insect cell-based VLPs as quadrivalent seasonal influenza vaccine candidates. The results presented herein shed light on the expression, characterization, stability, and immunogenicity of these VLPs, providing a valuable evaluation into their utility in the development of seasonal flu vaccines. These findings are in alignment with current research in the field. In our study, we successfully generated influenza A/H1N1, A/H3N2, B/Yamagata, and B/Victoria VLPs in High Five cells co-expressing HA, NA, and M1 proteins. Our findings, as confirmed by Western blot, SDS-PAGE, and TEM analysis, are consistent with previous studies [14,24]. In our study, we harnessed the High Five cell line to produce VLPs. These cells were chosen due to their co-expression of essential proteins (HA, NA, and M1), enabling the attainment of a substantial protein yield after baculovirus infection [14,24,25]. Indeed, removing the baculovirus residual from the concentrated VLPs produced by BEVS remains a significant challenge. Our pilot study was a small-scale production that employed simple purification procedures, and we measured the ratio between HA protein and total protein, which met the purity criteria of the WHO’s inactivated influenza vaccine guidelines [20]. According to the package insert of Flublok, baculovirus residual can be detected in the commercial Flublok vaccine. Moreover, U.S. FDA guidelines do not require quantification of baculovirus in recombinant vaccines. However, upon purifying VLPs, we observed significant variations in HA yields among the four influenza VLPs. The highest HA yield was achieved for B/Victoria (10.6 mg/L), followed by A/H3N2 (7.8 mg/L), A/H1N1 (6.6 mg/L), and B/Yamagata (2.4 mg/L)—findings comparable with those of similar productivity experiments [26,27]. In a parallel study conducted by Buffin and colleagues [28], VLPs were produced in mammalian cells (293 T) by co-expressing HA, NA, and M1 proteins, resulting in a maximum HA yield of 1.32 mg/L. Notably, when compared to this mammalian cell-based system, our insect cell-based VLPs exhibited significantly higher HA protein yields.

For development of a successful vaccine, the stability of a vaccine antigen is a critical factor. A previous study investigating the impact of temperature on influenza VLPs revealed that higher temperatures led to a significant reduction in HA activity and antigenicity within just one month, while lower temperatures, especially 4 °C, maintained stability over the same period [29]. Commercial protein vaccines are typically stored under regular cold chain (2–8 °C) to preserve their effectiveness and potency [30]. Furthermore, it was observed that the antigenicity of influenza VLPs significantly declined under acidic pH conditions [31]. Therefore, developing a suitable formulation to enhance the stability of influenza VLPs could prolong the vaccine shelf life and reduce vaccine wastage. In our study, we assessed the feasibility of using PBS and trehalose to store A/H1N1, A/H3N2, B/Yamagata, and B/Victoria VLPs at 4 °C and −20 °C. Our findings indicate that trehalose effectively preserved HA activity and HA protein concentration at both 4 °C and −20 °C, with particularly remarkable stability observed at 4 °C. The observed degradation of VLPs in PBS contrasted with the sustained stability in trehalose over time, which is consistent with earlier stability studies. A prior study by Lynch et al. found that HIV-1 Pr55^gag^ VLPs in PBS failed to maintain structural integrity even at very low temperatures like −70 °C, but 15% trehalose successfully maintained the stability at −20 °C over 12 months [32]. Other studies have also demonstrated the suitability of trehalose in efficiently maintaining the functional activities and structural integrity of influenza VLP [33,34,35,36].

In the mouse study, we explored the potential of quadrivalent VLPs formulations comprising A/H1N1, A/H3N2, B/Yamagata, and B/Victoria VLPs and compared the results with two commercial vaccines. The data suggested that quadrivalent VLPs were effective in eliciting a robust immune response against all vaccine antigens. Previous studies have demonstrated that VLPs expressing key proteins such as HA, NA, and M1 from respective influenza strains are highly immunogenic and can protect animals against lethal challenges [37,38,39]. In our study, we observed that the HAI and NT antibody responses of quadrivalent VLPs varied across the vaccine strains. Among the type A VLPs, the HAI titer was higher against A/H3N2 compared to A/H1N1, while in type B, Yamagata-VLPs exhibited higher titers than the Victoria-VLPs. This trend mirrors findings in clinical trials involving elderly populations, where trivalent egg-based inactivated virus vaccines and recombinant HA protein-based vaccines consistently showed favorable antibody responses toward the A/H3N2 virus over the A/H1N1 and type B viruses [40,41,42,43,44].

In our study, we conducted a comprehensive analysis of HAI and NT antibody responses elicited by VLP, recombinant HA, and egg-based vaccine antigens. To the best of our knowledge, this study represents the first set of head-to-head data comparing HAI and NT antibody responses, incorporating three distinct vaccine production platforms. Notably, the VLP vaccine antigens tend to induce more favorable HAI and NT antibody results compared to the recombinant HA vaccine group. A prior investigation highlighted the adjuvant properties of the live baculovirus content in BEVS-produced VLPs, enhancing cell-mediated immunity [45]. More recent data from phase III clinical trials on a plant-based influenza VLP vaccine demonstrated quantitatively enhanced antibody responses [46]. Surprisingly, the Flublok vaccine group exhibited no detectable HAI response against the B/Victoria virus. This result may be attributed to the comparatively lower B/Victora HA protein content in the Flublok vaccine formulation (Table 2). Previously published clinical study data also revealed that the Flublok vaccine is a relatively weak immunogen compared to the other three vaccine antigens [47]. In contrast, the egg-based Vaxigrip vaccine group induced modest HAI antibodies against the A/H1N1 and A/H3N2 viruses. Numerous studies have suggested that during the propagation of vaccine viruses in ECE, there is a tendency for antigenic drift, leading to vaccine mismatch and reduced HAI antibody production [7,48,49,50].

In summary, the findings underscore the potential of insect cell-based VLP vaccines as promising candidates for developing quadrivalent seasonal influenza vaccines. The robust immune responses observed in preclinical studies lay the foundation for further challenge studies in mice and ferrets.

## Figures and Tables

**Table 1:** List of influenza proteins included in the plasmids that were used in the quadrivalent VLP productions.

Type of Influenza Virus	Subtype or Lineage	Type of Protein	Strains *	GenBank and GISAID Accession Number
A	H1N1	HA	A/Hawaii/70/2019	MT738421.1
		NA	A/Hawaii/70/2019	MT738415.1
		M1	A/Hawaii/70/2019	MT738417.1
	H3N2	HA	A/Minnesota/41/2019	EPI1487157
		NA	A/Minnesota/41/2019	EPI1487156
		M1	A/Minnesota/41/2019	EPI1487152
B	Yamagata	HA	B/Brisbane/09/2014	EPI539769
		NA	B/Brisbane/09/2014	EPI544258
		M1	B/Brisbane/09/2014	EPI630126
	Victoria	HA	B/Darwin/7/2019	EPI1434729
		NA	B/Brisbane/63/2014	EPI646564
		M1	B/Brisbane/63/2014	EPI630121

* HA, NA, and M1 nucleotide sequences of CVV were chosen according to the WHO recommendations for influenza vaccine formulations for the 2020–2021 season.

**Table 2:** Assessment of strain specific HA protein concentration in commercial vaccines.

Vaccine Name	HA Protein Concentration (µg/mL)				Mean HA Concentration/Strain (µg/mL)
	A/H1N1	A/H3N2	B/Yamagata	B/Victoria	
Flublok	198	197	192	173	190
Vaxigrip	60	43	57	53	53

**Table 3:** HA yield of seasonal influenza VLPs produced in insect cells.

Virus Subtype	HA Titer before Purification (HAU/50 µL)	HA Titer after Purification (HAU/50 µL)	HA Content after Purification (µg/mL)	Total Protein Content after Purification (µg/mL)	HA Content/Total Protein (%)	HA Yield (µg) in 40 mL after Purification (mg/L) ^1^
A-H1N1	128	5120	220	929	23.7	264 (6.6)
A-H3N2	N/A ^2^	N/A ^2^	260	592	43.9	312 (7.8)
B-Yam	512	4096	80	443	18.1	96 (2.4)
B-Vic	1024	16,384	353	950	37.1	423.6 (10.6)

^1^ Estimated HA yield (mg/L) was based on the HA content (µg/mL) and volume (1.2 mL) after purification using 40 mL working volume and calculated by converting working volume from 40 mL to one liter. ^2^ Hemagglutination assay is not suitable for quantifying HA protein of recent influenza H3N2 viruses [21,22,23].

**Table 4:** Stability of the VLPs in PBS at 4 °C.

Name of the VLP	HAU/50 µL		HA Concentration (µg/mL)	
	Initial Titer	After 3 Months	Initial Concentration	After 3 Months
		4 °C		4 °C
Yamagata-VLPs	10,240	5120	77	55
Victoria-VLPs	20,480	10,240	165	110

**Table 5:** Stability of the VLPs in 20% trehalose at two different storage temperatures.

Name of the VLP	HAU/50 µL			HA Concentration (µg/mL)		
	Initial Titer ^1^	After 12 Months		Initial Concentration	After 12 Months	
		4 °C	−20 °C		4 °C	−20 °C
H1N1-VLPs	5120	2560	5120	94	84	87
H3N2-VLPs	N/A ^2^	N/A	N/A	262	217	243
Yamagata-VLPs	10,240	5120	5120	81	73	N/A ^3^
Victoria-VLPs	20,480	10,240	20,480	160	138	157

^1^ VLPs were harvested at 72 h post infection of the High Five cells. ^2^ Hemagglutination assay is not suitable for quantifying HA protein of recent influenza H3N2 viruses [21,22,23]. ^3^ HA concentration was not determined because samples were aggregated.

## Data Availability

The data presented in this study are available on request from the corresponding author.
